# Albumin-corrected anion gap as a predictor of 28-day mortality in acute respiratory distress syndrome: A machine learning-based retrospective study

**DOI:** 10.1371/journal.pone.0336662

**Published:** 2025-11-20

**Authors:** Qiudie Liu, Mengqi Zhang, Daoxin Wang

**Affiliations:** Department of Respiratory and Critical Care Medicine, The Second Affiliated Hospital of Chongqing Medical University, Chongqing, People’s Republic of China; University of Health Sciences, Beyhekim Training and Research Hospital, TÜRKIYE

## Abstract

**Background:**

Acute Respiratory Distress Syndrome (ARDS) remains a critical condition associated with high mortality rates, prolonged hospitalization, and reduced quality of life despite advances in critical care. The albumin-corrected anion gap (ACAG), an emerging biomarker reflecting acid-base disturbances, has been linked to poor outcomes in various critical illnesses. However, its prognostic value for mortality in ARDS patients remains unexplored.

**Methods:**

This retrospective study analyzed data from ARDS patients admitted to intensive care units (ICUs) in the MIMIC-IV database. Patients were stratified into quartiles (Q1–Q4) based on ACAG levels. The association between ACAG and 28-day all-cause mortality was comprehensively evaluated using restricted cubic splines, Kaplan–Meier survival analysis, and Cox proportional hazards regression. We employed the Boruta algorithm and LASSO (Least Absolute Shrinkage and Selection Operator) regression to identify key predictive factors. Six machine learning algorithms were used to develop predictive models, with performance assessed by the area under the ROC curve (AUC).

**Results:**

Higher ACAG levels were significantly associated with increased 28-day mortality risk in ARDS patients (P < 0.001). ACAG remained independently associated with 28-day all-cause mortality after comprehensive adjustment for confounders, with a hazard ratio (HR) of 1.04 (95% CI 1.01–1.07, P = 0.003) Subgroup analysis demonstrated that this association persisted across most demographic and clinical subgroups, with significant interactions observed only for myocardial infarction and malignancy (P for interaction < 0.05). Feature selection using Boruta and LASSO analyses consistently identified ACAG as a key predictor. Among the six machine learning models evaluated, the random forest (RF) algorithm demonstrated superior performance with an AUC of 0.73.

**Conclusions:**

Higher ACAG levels are independently associated with increased 28-day all-cause mortality in patients with ARDS. ACAG is a promising predictor of short-term mortality and may guide risk stratification in clinical practice.

## Introduction

Acute Respiratory Distress Syndrome (ARDS) is a life-threatening condition characterized by diffuse alveolar damage, noncardiogenic pulmonary edema, and a severe inflammatory response, leading to impaired gas exchange and respiratory failure [1–3]. Despite advancements in critical care management, including optimized ventilatory strategies and supportive therapies, ARDS remains associated with high mortality rates of 35%–46%, imposing a significant burden on healthcare systems worldwide [4]

Epidemiological data highlight the significant prevalence of ARDS in intensive care settings. A 6-month prospective study at a UK University Hospital ICU found that 43 of 344 admitted patients (12.5%) developed ARDS, consistent with the reported incidence range of 2.5%–19% [4]. A large international cohort study, reported that ARDS accounted for 10.4% of ICU admissions and 23.4% of patients requiring mechanical ventilation, with a period prevalence of 30.0% mild, 46.6% moderate, and 23.4% severe ARDS [5]. These statistics underscore the need for early and accurate prognostic assessment to improve outcomes and reduce medical costs.

Recent research has focused on identifying prognostic biomarkers for ARDS, including inflammatory markers and indicators of alveolar epithelial or endothelial injury. However, these markers often reflect only specific pathological aspects of ARDS and may not capture the comprehensive disease burden [6,7], Traditional tools like the Acute Physiology Score III (APS III) and Sequential Organ Failure Assessment (SOFA) scores are widely used to gauge disease severity and mortality risk [8]. However, their complexity, requirement for multiple variables, and inconsistent performance in certain patient subgroups limit their practical utility in resource-constrained settings. Thus, there remains an urgent need for simple, reliable, and complementary prognostic tools to improve risk stratification, especially given the variability in healthcare practices across regions, where universally applicable biomarkers are essential.

Metabolic acidosis is a common complication in ARDS and may reflect the degree of systemic hypoxia and inflammation [9–12]. The anion gap (AG), a routinely measured biomarker, quantifies the difference between unmeasured serum cations and anions, aiding in diagnosing acid-base imbalances and identifying causes of metabolic acidosis [13,14]. However, hypoalbuminemia, a common condition in critically ill patients, can significantly affect AG calculations, potentially leading to false negatives in detecting metabolic acidosis [15,16]. To overcome the limitations of conventional AG, the albumin-corrected anion gap (ACAG) was developed, adjusting for serum albumin levels to provide a more accurate reflection of acid-base status [17].

Previous studies have demonstrated the prognostic value of ACAG in various critical care settings, including septic patients with diabetes mellitus, congestive heart failure, and acute kidney injury (AKI) [18–20]. Compared with the conventional anion gap (AG), ACAG consistently shows superior predictive performance. For example, in ICU patients with asthma, Wang et al. reported that ACAG predicted 30-day mortality more accurately than AG, with areas under the curve (AUCs) of 0.703 versus 0.642 respectively, using an optimal cut-off of 20.38 mmol/L [21]. Similarly, in patients with acute myocardial infarction, elevated ACAG was independently associated with all-cause mortality (HR 1.423, 95% CI 1.206–1.678), and its AUC for predicting 360-day mortality (0.651) exceeded that of AG (0.609), further supporting the superior prognostic utility of ACAG over AG in diverse critical care settings [22]. Other investigations have also confirmed ACAG’s predictive advantage over conventional AG in various conditions [23].

In the context of ARDS specifically, Li et al. demonstrated that elevated AG was associated with 90-day mortality (HR 1.06, 95% CI 1.02–1.10); however, this study did not account for hypoalbuminemia, potentially underestimating the true extent of metabolic acidosis in this population [24]. Given that ARDS is frequently accompanied by both metabolic acidosis and hypoalbuminemia, the conventional AG may provide an incomplete assessment of acid-base status. Therefore, ACAG may offer superior prognostic value in ARDS patients, though this specific application remains unexplored. Our study aims to address this gap by evaluating ACAG as a predictor of 28-day mortality in ARDS patients. We investigated the relationship between ACAG and 28-day mortality and developed predictive models integrating ACAG with other clinical parameters using machine learning (ML) approaches. By establishing the prognostic value of ACAG in ARDS, our findings provide clinicians with a simple and readily available tool for risk stratification and outcome assessment in this high-mortality population.

## Methods

### Data source and study population

This is a retrospective study based on the clinical data of patients with ARDS from MIMIC-IV version 2.2, which includes information on over 65,000 ICU admissions and more than 200,000 emergency department admissions at Beth Israel Deaconess Medical Center (BIDMC) in Boston, Massachusetts, between 2008 and 2019 [25]. Patient confidentiality is maintained through de-identification (real data replaced with arbitrary numbers), eliminating the need for ethical approval and informed consent. The author accessed the database after completing the CITI Program (certificate #62816135). Data extraction and analysis procedures are detailed below, allowing replication by researchers with MIMIC-IV access.

**Fig 1:**
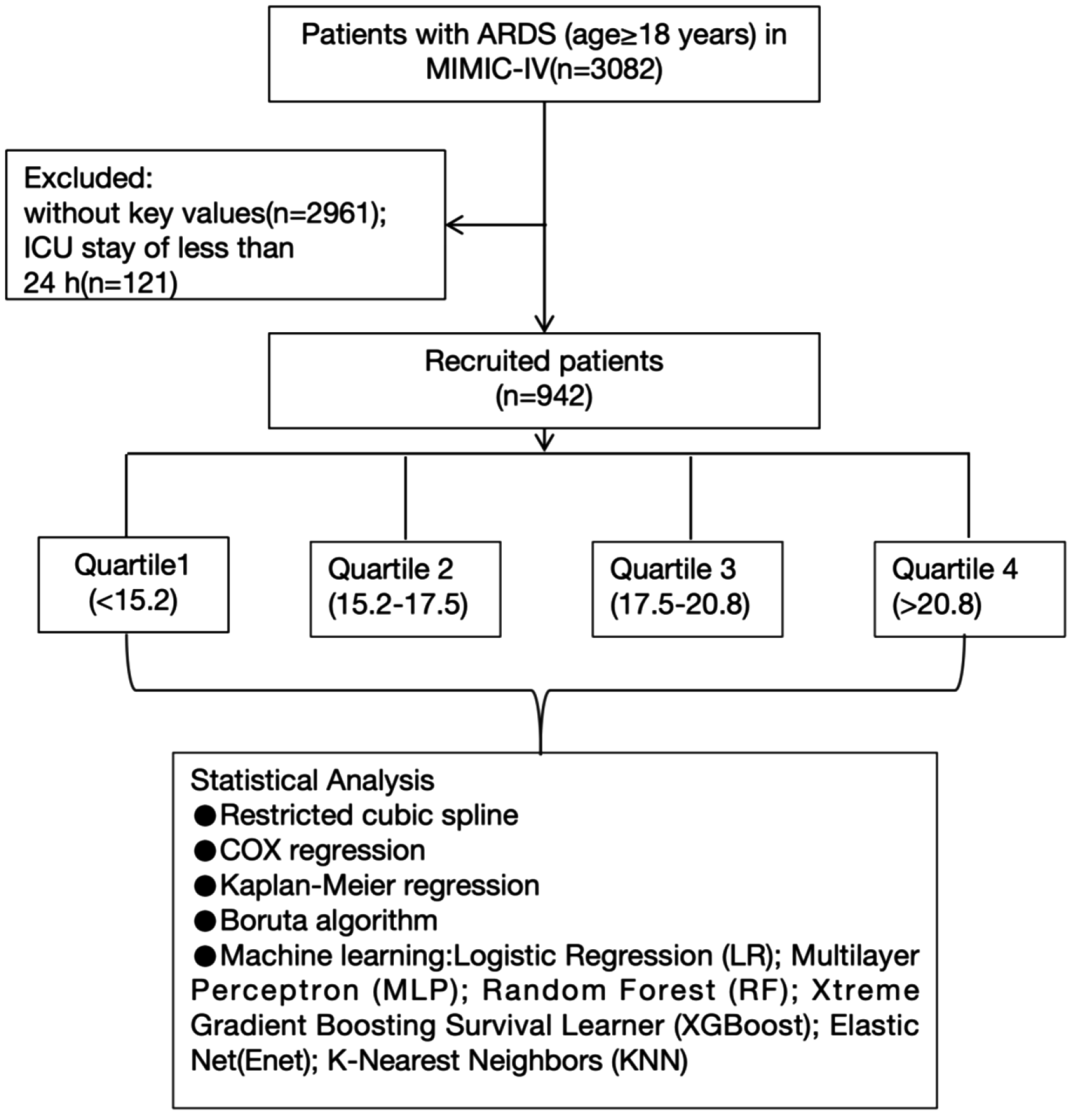
Selection of the study population from the MIMIC-IV database.

Admission information for ARDS patients was identified if they met the ICD-10 code J80 (ARDS) or fulfilled the Berlin definition [26]. The Berlin definition includes the following criteria: (1) acute onset of respiratory symptoms; (2) bilateral opacities on chest imaging; (3) a partial pressure of arterial oxygen (PaO₂) to fraction of inspired oxygen (FiO₂) ratio < 300 mmHg with a minimum positive end-expiratory pressure (PEEP) ≥ 5 cmH2O; and (4) absence of cardiac failure. Patients were excluded if they met any of the following conditions: (1) missing anion gap or albumin values in the first laboratory test; (2) ICU stay < 24 hours; (3) multiple ICU admissions (only the first admission was considered); or (4) age < 18 year. Ultimately, this study included 942 patients (Fig 1).

### Data extraction

Data was extracted using Navicat software. Characteristics were collected at the time of the patients’ admission including age, gender, race/ethnicity. Vital signs: Heart rate, mean blood pressure (MBP), body temperature, pulse oxygen saturation (SpO₂). Comorbidities: Diabetes, liver disease, renal disease, cerebrovascular disease, sepsis, myocardial infarction, and malignancy. Laboratory parameters: Anion gap (AG), albumin, white blood cell count (WBC), hemoglobin, platelet count, red cell distribution width (RDW), mean corpuscular volume (MCV), bicarbonate, blood urea nitrogen (BUN), creatinine, sodium, potassium, chloride, glucose, absolute neutrophil count, absolute lymphocyte count, absolute monocyte count, and absolute eosinophil count. Interventions: Renal replacement therapy (RRT) and vasopressor use. Severity scores: Sequential Organ Failure Assessment (SOFA) score and Acute Physiology Score III (APS III).

The ACAG value was calculated using the formula: ACAG = AG + [4.4– albumin (g/dL)] × 2.5 [17,27]. Missing data were handled using multiple imputation by chained equations (MICE) in R. A detailed summary of missingness for each variable is provided in Table S2 in S1 File. Variables with more than 25% missing values were excluded from the analysis. For the remaining variables, five imputed datasets were generated, and the estimates from each dataset were combined using Rubin’s rules to obtain pooled effect estimates and standard errors. MICE was chosen because it flexibly handles both continuous and categorical variables, preserves sample size, and yields valid estimates by incorporating uncertainty in the imputation process.

### Sampling size

The sample size was calculated according to the cross-sectional study sample size formula [28].


$$n=\frac{Z_{1-\frac{\alpha }{2}}^{2}p(1-p)}{d^{2}}$$

Where α = 0.05 (Z = 1.96), d = 0.05, and p represents the prevalence of the primary outcome. Based on previous studies, the mortality of ARDS patients was estimated at 46% [4]. The required sample size was therefore 381. In the present study, 942 patients were included, exceeding the minimum required sample size, indicating that the study had adequate statistical power and representativeness.

### Groups and clinical outcome

In line with previous research, ACAG levels > 20 mmol/L are generally considered high in clinical practice, while values between 12–20 mmol/L are regarded as normal [29]. In our study, to better reflect the distribution of ACAG within the cohort, patients were stratified into quartiles (Q1 < 15.2, Q2 15.2–17.5, Q3 17.5–20.8, Q4 > 20.8). The primary clinical outcome was the 28-day mortality after ICU admission.

### Statistical analysis

Normally distributed continuous variables are presented as the means (standard deviations [SDs]) and were analyzed using analysis of variance (ANOVA). Nonnormally distributed variables were analyzed using the Mann-Whitney U test or the Kruskal-Wallis test. Categorical variables are expressed as numbers and percentages and were analyzed using the χ2 test or Fisher’s exact test. Kaplan-Meier survival curves were constructed to compare 28-day mortality across ACAG quartiles, with differences assessed using the log-rank test. Restricted cubic spline (RCS) analysis was performed to examine the potentially non-linear relationship between ACAG as a continuous variable and 28-day mortality. Multivariate Cox proportional hazards regression was used to evaluate the independent association between ACAG and 28-day mortality after adjusting for potential confounders. Hazard ratios (HR) and 95% confidence intervals (95% CI) were reported. We constructed three models with progressive adjustment: Model I: Unadjusted model; Model II: Adjusted for demographics and clinical characteristics (age, gender, race, vital signs, and comorbidities); Model III: Fully adjusted model including all variables in Model II plus laboratory parameters, severity scores, and interventions. Subgroup analyses were conducted to assess whether the association between ACAG and mortality was consistent across different patient subgroups, including age, gender, comorbidities, and interventions.

To identify key predictive features, we employed two complementary feature selection methods: Boruta Algorithm: A wrapper algorithm based on random forest that determines feature importance by comparing original attributes with their randomly permuted copies; LASSO (Least Absolute Shrinkage and Selection Operator) Regression: A regularization technique that performs variable selection by penalizing the absolute size of the regression coefficients. Variables selected by both methods were included in the final prediction models. Multicollinearity among selected features was assessed using variance inflation factors (VIF), with values < 5 considered acceptable. The dataset was randomly divided into a training set (70%, n = 658) and a validation set (30%, n = 284). Six machine learning algorithms were implemented: Logistic Regression (LR), K-Nearest Neighbors (KNN), Random Forest (RF), Extreme Gradient Boosting (XGBoost), MLP (Multilayer Perceptron), Enet (Elastic Net) algorithms were employed to assess the 28-day death risk in ARDS patients, respectively. The ROC curves and AUCs were utilized to assess model performance. Decision curve analysis (DCA) was implemented to evaluate clinical effectiveness, while calibration curves were employed to measure the accuracy of absolute risk predictions. For the best-performing model, we further explored feature importance using partial dependence plots (PDPs) and SHAP (SHapley Additive exPlanations) values to understand how each feature contributes to the predictions. The detailed of model development details is provided in Table S2 in S1 File.

All statistical analyses were conducted using R software (version 4.4.5), with a p-value < 0.05 considered statistically significant.

## Results

### Participant characteristics

After applying the inclusion and exclusion criteria, a total of 942 eligible patients with ARDS were included in our analysis (Fig 1). Table 1 presents the baseline characteristics of patients stratified by ACAG quartiles. Patients in higher ACAG quartiles exhibited significantly higher heart rates, body temperatures, white blood cell counts, platelet counts, red cell distribution width (RDW), serum creatinine, blood urea nitrogen (BUN), and lactate levels. They also had higher SOFA and APS III scores, indicating greater disease severity. Additionally, these patients had higher prevalence of diabetes, malignant cancer, myocardial infarction, acute kidney injury (AKI), and sepsis, and were more likely to require renal replacement therapy (RRT). Conversely, they had lower levels of bicarbonate, albumin, and chloride, consistent with more severe metabolic acidosis. The overall 28-day mortality rate in the cohort was 21.3% (201/942). Notably, mortality rates increased progressively across ACAG quartiles: 18.8% in Q1, 16.0% in Q2, 21.5% in Q3, and 29.1% in Q4 (P < 0.001)

**Table 1:** Patient demographics and baseline characteristics.

Variables	Total (n = 942)	Q1 (n = 250)	Q2 (n = 225)	Q3 (n = 237)	Q4 (n = 230)	p
Age (year)	66.00 [54.00, 76.00]	66.50 [55.25, 76.00]	67.00 [56.00, 77.00]	67.00 [56.00, 76.00]	62.50 [51.00, 73.00]	0.004
**Gender, n (%)**						
Female	397 (42.1)	102 (40.8)	101 (44.9)	105 (44.3)	89 (38.7)	0.483
Male	545 (57.9)	148 (59.2)	124 (55.1)	132 (55.7)	141 (61.3)	
Race, n(%)						
OTHER	356 (37.8)	82 (32.8)	86 (38.2)	89 (37.6)	99 (43.0)	0.146
WHITE	586 (62.2)	168 (67.2)	139 (61.8)	148 (62.4)	131 (57.0)	
**Vital signs**						
Heart rate	86.89 [77.01, 99.50]	83.56 [74.95, 93.05]	85.62 [76.14, 97.50]	87.88 [77.50, 99.96]	93.69 [81.18, 104.42]	<0.001
SpO2	97.65 [96.04, 98.82]	97.75 [96.23, 98.90]	97.65 [96.27, 98.71]	97.44 [95.94, 98.81]	97.91 [96.06, 98.97]	0.531
MBP	74.46 [69.06, 81.55]	74.62 [68.45, 81.52]	75.47 [70.24, 81.56]	73.12 [68.48, 80.38]	74.80 [69.09, 82.85]	0.39
Temperature (°C)	36.93 [36.66, 37.31]	36.91 [36.62, 37.22]	36.91 [36.65, 37.25]	36.94 [36.64, 37.30]	36.95 [36.72, 37.45]	0.108
**Laboratory tests**						
Hemoglobin (g/L)	12.03 (2.38)	12.53 (2.05)	11.97 (2.32)	11.78 (2.36)	11.82 (2.72)	<0.001
Platelets (10^9^/L)	211.00 [152.00, 279.50]	209.00 [156.25, 265.00]	215.00 [153.00, 275.00]	216.00 [153.00, 298.00]	201.50 [144.50, 281.50]	0.816
WBC (10^9^/L)	9.45 [6.60, 14.00]	7.60 [6.00, 10.40]	8.60 [6.30, 11.50]	10.50 [6.90, 15.20]	13.05 [9.00, 17.85]	<0.001
PaO2/FiO2	172.25 [103.00, 237.38]	175.50 [109.25, 241.50]	172.50 [103.00, 237.50]	172.00 [106.00, 228.00]	164.14 [96.00, 242.00]	0.607
MCV	92.00 [88.00, 97.00]	91.00 [87.00, 96.00]	92.00 [88.00, 96.00]	91.00 [88.00, 97.00]	93.00 [88.00, 98.00]	0.079
Eosinophils count (10^9^/L)	0.07 [0.01, 0.17]	0.11 [0.04, 0.20]	0.10 [0.04, 0.20]	0.05 [0.01, 0.15]	0.03 [0.00, 0.09]	<0.001
Lymphocytes count(10^9^/L)	1.20 [0.73, 1.83]	1.37 [0.88, 2.01]	1.30 [0.80, 1.85]	1.09 [0.68, 1.68]	1.09 [0.62, 1.72]	<0.001
Monocytes count (10^9^/L)	0.50 [0.32, 0.80]	0.43 [0.31, 0.62]	0.49 [0.34, 0.70]	0.53 [0.30, 0.83]	0.62 [0.35, 0.97]	<0.001
Neutrophils count (10^9^/L)	6.82 [4.24, 11.88]	5.38 [3.60, 7.56]	5.80 [3.91, 9.72]	7.85 [4.52, 12.24]	10.02 [6.30, 14.53]	<0.001
Neutrophils (%)	76.00 [65.90, 85.00]	71.10 [60.47, 81.20]	72.80 [64.00, 83.10]	78.20 [67.10, 86.40]	82.80 [74.00, 87.00]	<0.001
Albumin (mg/dL)	3.70 [3.10, 4.10]	4.00 [3.50, 4.30]	3.80 [3.30, 4.20]	3.60 [2.90, 4.00]	3.30 [2.70, 3.88]	<0.001
RDW (%))	14.40 [13.50, 15.80]	13.90 [13.20, 15.20]	14.40 [13.50, 15.90]	14.60 [13.70, 16.00]	14.70 [13.70, 16.40]	<0.001
Anion gap (mmol/L)	16.00 [13.00, 19.00]	12.00 [11.00, 14.00]	15.00 [14.00, 16.00]	17.00 [16.00, 18.00]	21.00 [19.25, 24.00]	<0.001
BNU (mmol/L)	21.00 [15.00, 33.00]	18.00 [13.00, 24.00]	19.00 [13.00, 28.00]	24.00 [16.00, 37.00]	31.50 [18.00, 57.00]	<0.001
Scr (mg/dL)	1.10 [0.80, 1.60]	0.90 [0.80, 1.20]	1.00 [0.80, 1.30]	1.10 [0.80, 1.60]	1.60 [1.00, 3.10]	<0.001
Chloride (mmol/L)	102.00 [99.00, 105.00]	103.00 [101.00, 105.00]	103.00 [100.00, 105.00]	102.00 [98.00, 106.00]	101.00 [96.00, 105.00]	<0.001
Sodium (mmol/L)	138.00 [135.00, 141.00]	139.00 [137.00, 141.00]	139.00 [136.00, 141.00]	138.00 [134.00, 141.00]	137.00 [133.00, 141.00]	0.014
Potassium (mmol/L)	4.10 [3.70, 4.60]	4.10 [3.80, 4.38]	4.10 [3.80, 4.60]	4.20 [3.80, 4.80]	4.25 [3.60, 5.10]	0.112
Bicarbonate (mmol/L)	24.00 [21.00, 27.00]	28.00 [26.00, 29.00]	25.00 [23.00, 27.00]	23.00 [20.00, 25.00]	19.00 [16.00, 22.00]	<0.001
**Score**						
SOFA	8.00 [5.00, 11.00]	7.00 [4.00, 9.75]	7.00 [4.00, 10.00]	8.00 [5.00, 11.00]	8.00 [6.00, 12.00]	<0.0001
APSIII	53.00 [40.00, 71.75]	49.00 [35.00, 65.00]	49.00 [37.00, 65.00]	55.00 [42.00, 73.00]	61.00 [46.00, 80.00]	<0.0001
Charlson comorbidity index	5.00 [3.00, 7.00]	5.00 [3.00, 7.00]	5.00 [4.00, 7.00]	5.00 [4.00, 7.00]	5.00 [3.00, 7.00]	0.034
**Comorbidities**						
Diabetes without comorbidity, n (%)	243 (25.8)	62 (24.8)	60 (26.7)	49 (20.7)	72 (31.3)	0.068
Diabetes with comorbidity, n (%)	91 (9.7)	23 (9.2)	21 (9.3)	21 (8.9)	26 (11.3)	0.398
Malignant cancer, n (%)	105 (11.1)	35 (14.0)	24 (10.7)	24 (10.1)	22 (9.6)	0.004
Myocardial infarction, n (%)	175 (18.6)	48 (19.2)	36 (16.0)	42 (17.7)	49 (21.3)	0.513
Severe liver disease, n (%)	124 (13.2)	35 (14.0)	28 (12.4)	32 (13.5)	29 (12.6)	0.952
AKI, n (%)	678 (72.0)	165 (66.0)	153 (68.0)	168 (70.9)	192 (83.5)	<0.001
Cerebrovascular disease, n (%)	126 (13.4)	31 (12.4)	26 (11.6)	39 (16.5)	30 (13.0)	0.421
Sepsis, n (%)	665 (70.6)	161 (64.4)	141 (62.7)	180 (75.9)	183 (79.6)	<0.001
**Therapy**						
RRT, n (%)	81 (8.6)	18 (7.2)	12 (5.3)	14 (5.9)	37 (16.1)	<0.001
vasopressor use, n (%)	369 (39.2)	94 (37.6)	71 (31.6)	96 (40.5)	108 (47.0)	<0.001
**Clinical outcomes**						
ICU stay(days)	8.25 (8.51)	7.32 (7.56)	8.04 (8.87)	7.91 (7.42)	9.81 (9.91)	0.01
Hospital stay (days)	16.30 (15.18)	14.87 (12.95)	16.63 (17.26)	15.66 (11.05)	18.20 (18.46)	0.09
Hospital mortality, n (%)	178 (18.9)	42 (16.8)	30 (13.3)	46 (19.4)	60 (26.1)	0.004
28-day mortality	201 (21.3)	47 (18.8)	36 (16.0)	51 (21.5)	67 (29.1)	<0.001

ACAG: Quartile 1 < 15.2, Quartile 2 (14.76–17.5), Quartile 3 (17.5–20.8), and Quartile 4 > 20.8.

Abbreviation: MBP, mean blood pressure; WBC, white blood cell count; RDW, red cell distribution width; MCV, mean corpuscular volume; BUN, blood urea nitrogen; Scr, surmue creatinine; RRT, Renal Replacement Therapy; SOFA, Sequential Organ Failure score; APS III, Acute Physiology Score III score; AKI, acute kidney injury.

### Kaplan-Meier survival curve analysis

**Fig 2:**
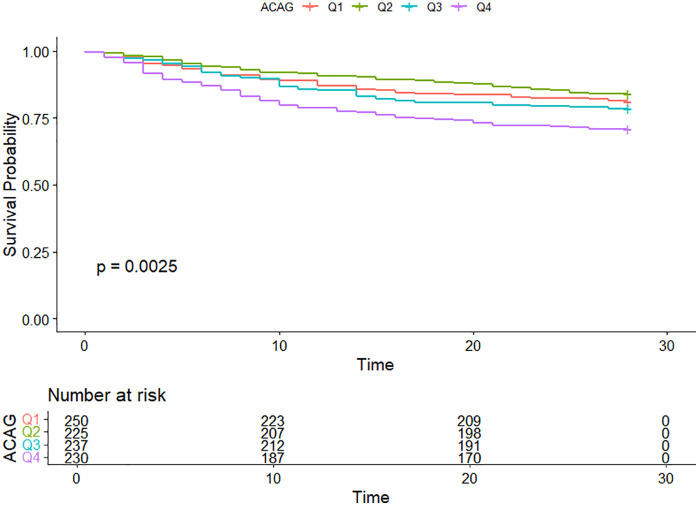
28-day KM survival curve. ACAG: Quartile 1 < 15.2; Quartile 2 (15.2–17.5); Quartile 3 (17.5–20.8), and Quartile 4 > 20.8.

During the 28-day follow-up period, 201 patients (21.3%) experienced mortality. Kaplan-Meier survival curves for 28-day mortality stratified by ACAG quartiles (Fig 2) demonstrated a significant separation between groups, with higher ACAG levels associated with poorer survival (log-rank test, P < 0.001).

### Association between ACAG and 28-day mortality and the subgroup analysis

**Fig 3:**
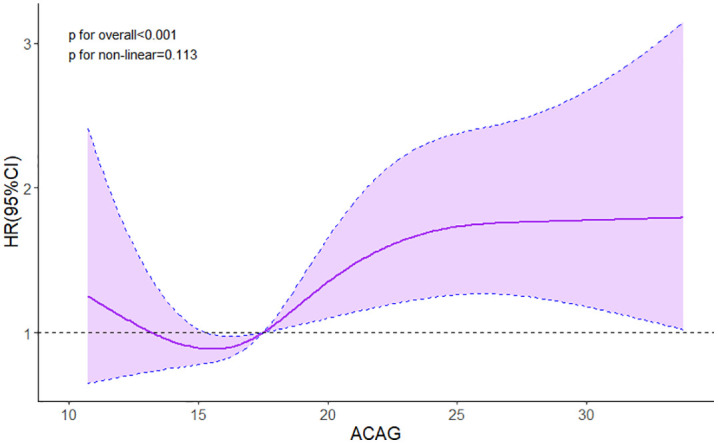
RCS analysis of 28-day all-cause mortality. Curves represent estimated hazard ratios, and shaded ribbons represent 95% confidence intervals. HR hazard ratio; CI confidence interval.

The relationship between ACAG and 28-day mortality was further examined using Cox proportional hazards regression models with different levels of adjustment (Table 2). Covariates included in the multivariable models were selected based on clinical relevance and previously reported prognostic importance in critically ill patients, in order to minimize potential confounding. Higher levels of ACAG were significantly associated with increased 28-day mortality in patients with ARDS. In the unadjusted model, we observed a significant association between ACAG and 28-day mortality in ARDS patients (HR:1.04, 95% CI 1.01–1.06, P = 0.003). Even in the fully adjusted model, ACAG persisted as an independent risk factor. When analyzed as quartiles variable, patients in the highest ACAG(Q4) quartile had a significantly higher risk of 28-day mortality compared to those in the lowest quartile(Q1) in the fully adjusted model (HR = 1.66; 95% CI: 1.14–2.42; p = 0.008). Moreover, there was a significant linear trend across quartiles (P for trend < 0.05), further supporting a dose-response relationship. Moreover, in the multivariable restricted cubic spline model (Fig 3), a linear association was observed between ACAG and the risk of 28-day mortality (P for nonlinear = 0.113, P for overall < 0.001).

**Table 2:** Cox proportional hazard regression analysis for 28-d mortality in patients with ARDS.

Variable	Model1	P value	Model2	P value	Model3	P value
	HR (95% CI)		HR (95% CI)		HR (95% CI)	
ACAG continuous	1.04 (1.01-1.06)	0.003	1.03 (1.01-1.06)	0.01	1.04 (1.01-1.07)	0.003
Categories						
Quartile 1						
Quartile 2	0.83 (0.54-1.28)	0.392	0.76 (0.49-1.17)	0.209	0.81 (0.52-1.25)	0.339
Quartile 3	1.16 (0.78-1.72)	0.463	1.02 (0.68-1.52)	0.92	1.06 (0.71-1.58)	0.784
Quartile 4	1.67 (1.15-2.43)	0.007	1.53 (1.05-2.24)	0.029	1.66 (1.14-2.42)	0.008
P for trend		0.001		0.007		0.05

Model 1 is the unadjusted model;

Model II adjusts for age, gender, race, heart rate, mean blood pressure (MBP), SpO2, temperature, myocardial infarction, cerebrovascular disease, diabetes, malignant cancer, severe liver disease, acute kidney injury (AKI), sepsis, and the Charlson comorbidity index;

Model III was adjusted for age, cerebrovascular disease, malignant cancer, and the Charlson comorbidity index, hemoglobin, platelet count, white blood cell count (WBC), creatinine, sodium, potassium, absolute eosinophils, absolute lymphocytes, absolute monocytes, bicarbonate, blood urea nitrogen (BUN), chloride, use of renal replacement therapy (RRT), PaO₂/FiO₂ ratio, and vasopressor use.

The restricted cubic spline analysis (Fig 3) confirmed a predominantly linear association between ACAG and the risk of 28-day mortality (P for nonlinearity = 0.113, P for overall association < 0.001), with the risk increasing progressively as ACAG levels rose above approximately 15 mmol/L.

### Subgroup analysis

**Fig 4:**
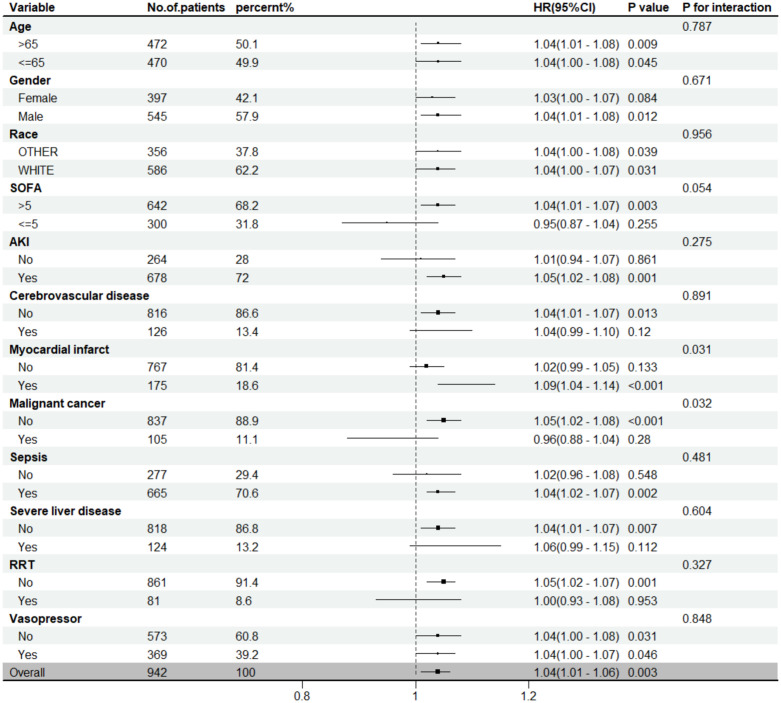
Subgroup forest plot for 28-day all-cause mortality.

Subgroup analyses across demographics, comorbidities, and therapeutic interventions generally demonstrated a consistent prognostic value of ACAG in ARDS patients. Notably, significant interactions were only observed in subgroups with myocardial infarction and malignant cancer, suggesting potential effect modification in these populations (Fig 4).

### Feature selection

**Fig 5:**
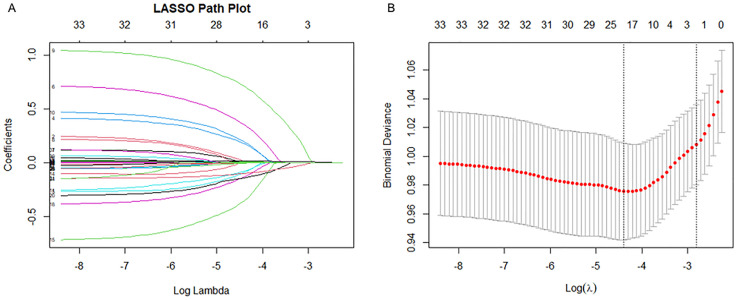
LASSO regression analysis results for 34 variables. (A) LASSO coefficient of variables. (B) Cross-validation curve.

**Fig 6:**
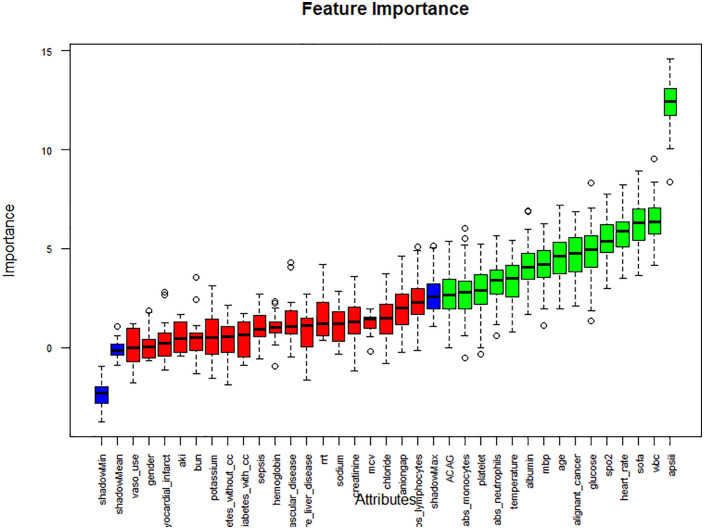
Variable selection procedure using the Boruta algorithm.

First, LASSO regression with fivefold cross-validation was applied to eliminate non-informative variables. The optimal penalty parameter (λ) minimizing cross-validation error was selected to balance model parsimony and generalizability (Fig 5A), resulting in 18 features with non-zero coefficients (Fig 5B). Subsequently, the Boruta algorithm, a random forest–based wrapper method, was employed to further evaluate feature relevance by comparing original predictors with permuted shadow features (Fig 6), confirming 15 variables as statistically significant. To ensure robustness and clinical interpretability, we retained the intersection of features consistently identified by both methods, yielding a final predictor set comprising ACAG, age, malignant cancer, APS III score, SOFA score, heart rate, body temperature, SpO₂, glucose, WBC count, absolute neutrophil count, and albumin. Variance inflation factor (VIF) analysis confirmed the absence of significant multicollinearity, with all VIF values < 5.

### Machine learning model development

**Fig 7:**
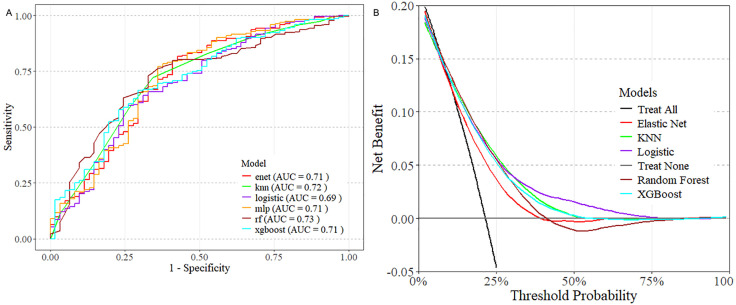
Establishment and validation of the machine learning prediction model. **A** ROC curve of the machine learning model. **B** Decision curve analysis (DCA). LR,Logistic Regression, KNN, K-Nearest Neighbors, RF,Random Forest, XGBoost,Extreme Gradient Boosting, MLP, Multilayer Perceptron, Enet, Elastic Net.

The performance of the six machine learning models in predicting 28-day mortality is presented in Fig 7A. The Random Forest (RF) algorithm demonstrated the best discrimination with an AUC of 0.73 in the validation set, followed by K-Nearest Neighbors (AUC = 0.72), Elastic Net (AUC = 0.71), Multilayer Perceptron (AUC = 0.71), XGBoost (AUC = 0.71), and Logistic Regression (AUC = 0.69). Decision curve analysis (Fig 7B) showed that all models provided substantial net benefit across a wide range of threshold probabilities, confirming their potential clinical utility. Calibration curves indicated good agreement between predicted and observed outcomes for the RF, LR, KNN, and XGBoost models (Fig S1 in S1 file). Taken together, the Random Forest algorithm performed best overall.

### Interpretability analysis

**Fig 8:**
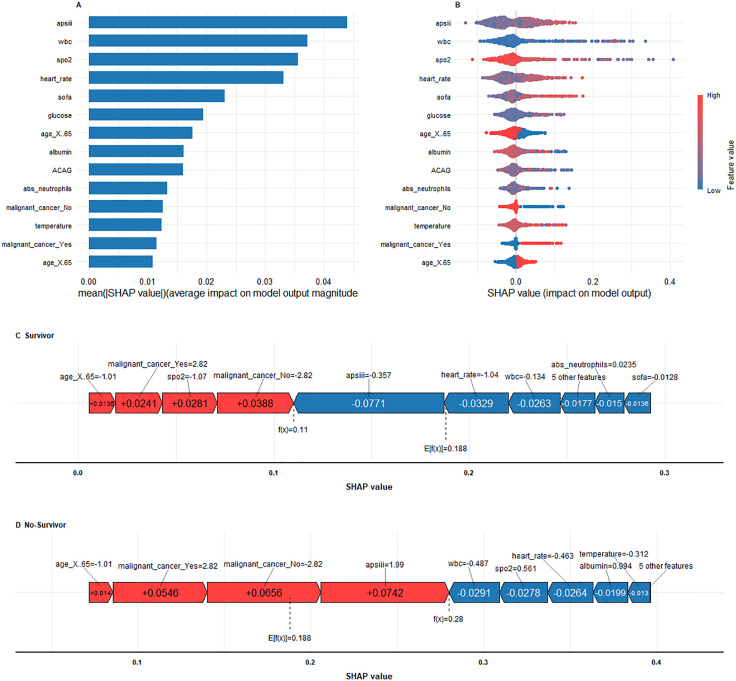
SHAP interprets the mode. (A) SHAP feature importance plot; (B) SHAP summary plot; (C, D) SHAP force plot.

To enhance interpretability, we employed SHAP analysis to examine both the global and individual-level contributions of predictive features in the Random Forest model, which achieved the highest overall performance. The SHAP-based feature importance ranking plot (Fig 8A) and swarm plot (Fig 8B) depict the global impact of each variable on model predictions. On the horizontal axis, SHAP values quantify both the magnitude and direction of each feature’s contribution to the predicted outcome, while the vertical axis ranks the variables according to their cumulative importance. Our analysis revealed that APS III score, SOFA score, WBC count, SpO₂, and ACAG were among the influential predictors.

Additionally, SHAP force plots were applied to visualize case-specific predictions (Fig 8C and 8D). These exemplary cases illustrate the model’s decision-making process for a survivor and a non-survivor. The visualization employs a dual-color coding scheme, with red denoting risk-enhancing factors (positive SHAP values) and blue representing protective factors (negative SHAP values). In the non-survivor case, the model predicted a substantially elevated 28-day mortality risk compared with the baseline population, driven by the predominance of risk-enhancing variables. Such individualized explanations provide clinicians with transparent insights into how different factors interact to shape mortality risk, thereby facilitating early recognition of clinical deterioration and enabling data-driven therapeutic decision-making in ARDS management.

## Discussion

This retrospective study comprehensively investigated the prognostic significance of the albumin-corrected anion gap (ACAG) in patients with acute respiratory distress syndrome (ARDS), and its association with 28-day all-cause mortality. Our results demonstrate that higher ACAG levels are independently associated with an increased risk of short-term mortality, with a 4% increase in mortality risk per 1 mmol/L increase in ACAG in our fully adjusted model. This relationship was validated through multiple analytical approaches, including restricted cubic splines (RCS), Kaplan–Meier survival analysis, Cox proportional hazards regression, and machine learning-based predictive modeling. These findings highlight ACAG as a promising, simple, and accessible biomarker for risk stratification in ARDS, with potential clinical utility in the intensive care unit (ICU) setting.

Acid–base balance plays a central role in maintaining physiological stability in critical illness [30]. Metabolic acidosis, the most common acid–base disturbance in critically ill patients, can be categorized into high anion gap (AG) and normal AG metabolic acidosis [10,31]. The AG has long been used as a diagnostic tool and has shown prognostic value in several conditions, including acute ischemic stroke and coronary artery disease [32,33]. However, since albumin constitutes a substantial portion of unmeasured anions, hypoalbuminemia, common in critically ill patients, can lead to a falsely low AG, potentially misguiding clinical assessment [34]. Hypoalbuminemia reflects both nutritional status and the acute phase response to inflammation, both of which have prognostic implications in critical illness. A secondary analysis of 794 septic patients with acute kidney injury undergoing continuous renal replacement therapy in Korea showed that each 1 g/dL increase in albumin level was independently associated with a 25% and 27% reduction in the risk of death at 28 and 90 d, respectively [35].

In ARDS, the pathophysiological mechanisms such as widespread endothelial injury, impaired oxygenation, and systemic hypoperfusion contribute to increased lactic acid production and accumulation, a key cause of high AG metabolic acidosis [2]. Additionally, liver and kidney dysfunction, which are common in ARDS and further impair acid clearance, can exacerbate acid–base imbalance [36,37]. Clinical evidence suggests that a greater degree of metabolic acidosis at admission, reflected by a lower base deficit, is associated with an increased risk of developing acute lung injury [37]. Given the limitations of conventional AG in the context of hypoalbuminemia, the albumin-corrected anion gap (ACAG) provides a more accurate reflection of acid–base disturbances [17]. Compared with AG alone, ACAG better reflects metabolic status and disease burden, supporting its role as a complementary prognostic biomarker in ARDS.

Our findings extend the existing evidence base by specifically demonstrating ACAG’s prognostic value in ARDS patients. This aligns with prior research linking elevated AG and ACAG to worse outcomes across various critical illnesses, including congestive heart failure, acute kidney injury, chronic obstructive pulmonary disease, sepsis, and asthma [18–20,38,39]. For instance, in acute respiratory failure, elevated ACAG has been linked to 28-day mortality [40].

Subgroup analysis in our study showed that the association between ACAG and mortality was consistent across most clinical subgroups, supporting its generalizability as a prognostic marker. However, significant interactions were observed in patients with myocardial infarction and malignancy, suggesting that comorbid conditions may modify the prognostic utility of ACAG. A recent large-scale retrospective cohort study demonstrated that ACAG was independently associated with an increased risk of in-hospital mortality in patients with acute myocardial infarction (AMI) [41]. The different strength of association in these subgroups highlights the need for disease-specific considerations when interpreting ACAG values and the potential for developing tailored risk prediction models for different patient populations.

A key strength of our study is the application of machine learning to identify important predictors and build predictive models. Variable selection techniques, including Boruta and least absolute shrinkage and selection operator (LASSO), consistently identified ACAG as a key prognostic feature. In the random forest model, which achieved the highest predictive accuracy (AUC = 0.73), ACAG ranked alongside APS III, SOFA, white blood cell (WBC) count, SpO₂, and heart rate, underscoring its potential to complement complex severity scores. Although predictive performance was moderate, ACAG is a simple, readily available marker requiring only two laboratory measurements (anion gap and albumin), which can be obtained rapidly at the bedside for early identification of high-risk patients. Integrating ACAG with established scores may enhance risk stratification and support timely clinical decision-making in the ICU, particularly in patients with hypoalbuminemia where traditional scores like APS III or SOFA may underestimate metabolic derangements [3,8]. This aligns with prior studies in other populations, such as AKI in heart failure, where combining ACAG with SOFA or APS III improved prognostic performance (AUCs 0.68–0.75) [42]. Similarly, in patients with AKI, ACAG outperformed the conventional anion gap and further enhanced the prognostic ability of the SOFA score (AUC = 0.662) [20].

ACAG provides particular value in patients with hypoalbuminemia, where conventional anion gap may underestimate metabolic acidosis severity, offering more accurate acid-base status assessment for precise clinical decision-making. Since ACAG calculation requires only routine laboratory measurements obtained at ICU admission, it can be rapidly implemented without additional testing burden across diverse healthcare settings, including resource-limited environments, while providing immediate prognostic information to guide early clinical decisions and optimize resource allocation.

Despite these promising findings, several limitations should be acknowledged. First, this was a single-center retrospective study based on data from a single tertiary care hospital in the United States, which may introduce potential selection bias and limit the generalizability of our findings to other settings, patient populations, or healthcare systems. Second, the study relied on a single database (MIMIC-IV), and external validation in independent cohorts was not performed. Third, although we adjusted for multiple potential confounders, residual confounding cannot be entirely ruled out due to the observational nature of the study; unmeasured variables such as genetic factors, pre-hospital treatment, or specific ventilator settings could influence both ACAG levels and mortality outcomes. Fourth, ACAG was assessed only at ICU admission, and dynamic changes over time were not evaluated. Serial measurements of ACAG could potentially provide additional prognostic information and better reflect the evolving metabolic status of critically ill patients. Fifth, while we demonstrated an association between ACAG and mortality, causality cannot be inferred. Finally, missing data were handled using multiple imputation by chained equations, which assumes missing at random; residual bias due to missing not at random cannot be entirely excluded.

## Conclusion

Elevated ACAG is an independent predictor of 28-day mortality in ARDS patients. As a readily available and easily interpretable parameter, ACAG may serve as a valuable adjunct to existing severity scores for early risk stratification in the ICU.

## Supporting information

S1 File Tables S1–S2 and Fig S1: missingness, model development detail, Calibration plot. (DOCX)
