# Supplementary material for: Albumin-corrected anion gap as a predictor of 28-day mortality in acute respiratory distress syndrome: A machine learning-based retrospective study
Source: PLoS One. 2025 Nov 20;20(11):e0336662. doi: 10.1371/journal.pone.0336662 (PMC12633864; doi:10.1371/journal.pone.0336662)
Supplement: S1 File — (DOCX) [file pone.0336662.s001.docx]

**Supplementary Material**

**Supplementary Table S1. Proportion of missing values for each variable.**

| Variable | Missing Percentage (%) |
| --- | --- |
| PaO2/FiO2 | 0.00 |
| Charlson index | 0.00 |
| SOFA | 0.00 |
| APSIII | 0.00 |
| Heart rate | 0.00 |
| MBP | 0.00 |
| Temperature (℃) | 7.54 |
| SpO2 | 0.00 |
| Eosinophils count (10^9^/L) | 23.35 |
| Lymphocytes count (10^9^/L) | 23.04 |
| Monocytes count (10^9^/L) | 23.35 |
| Neutrophils count (10^9^/L) | 23.35 |
| Neutrophils(%) | 23.35 |
| Hemoglobin | 0.42 |
| MCW | 0.53 |
| RDW(%) | 0.53 |
| Platelet (10^9^/L) | 0.74 |
| WBC (10^9^/L) | 0.42 |
| albumin (mg/dL) | 0.00 |
| anion gap (mmol/L) | 0.00 |
| BUN (mmol/L) | 0.11 |
| Scr (mmol/L) | 0.21 |
| sodium (mmol/L) | 0.00 |
| potassium (mmol/L) | 0.00 |
| bicarbonate (mmol/L) | 0.00 |
| chloride (mmol/L) | 0.00 |

Abbreviation: MBP, mean blood pressure; WBC, white blood cell count; RDW, red cell distribution width; MCV, mean corpuscular volume; BUN, blood urea nitrogen; Scr, surmue creatinine; RRT, Renal Replacement Therapy; SOFA, Sequential Organ Failure score; APS III, Acute Physiology Score III score.

**Supplementary Table S2. Model development detail.**

| Model | Parameter tuning (range) | Cross-validation strategy | Software packages |
| --- | --- | --- | --- |
| Random Forest (RF) | mtry (2–8); trees (200–400); min_n (30–50) | 5-fold stratified CV | tidymodels, randomForest, yardstick |
| Multilayer Perceptron (MLP) | hidden_units (15–24); penalty (10^-3 to 1); epochs (50–150) | 5-fold stratified CV | tidymodels, nnet, yardstick |
| Extreme Gradient Boosting (XGBoost) | mtry (2–6); tree_depth (3–5); min_n (5–10); learn_rate (10^-3 to 10^-2); loss_reduction (10^-2 to 1); sample_size (0–1) | 5-fold stratified CV with early stopping (stop_iter = 25) | tidymodels, xgboost, yardstick |
| Elastic Net (ENet) | mixture (0–1, ridge to lasso); penalty (10^-5 to 1) | 5-fold stratified CV | tidymodels, glmnet, yardstick |
| K-Nearest Neighbor (KNN) | neighbors (3–20); weight_func (rectangular, triangular); dist_power (1–2) | 5-fold stratified CV | tidymodels, kknn, yardstick |
| Logistic Regression(LR) | Not applicable | 5-fold stratified CV | tidymodels, glm, yardstick |

Note: Hyperparameter tuning was performed using grid or Latin hypercube search. All models were evaluated with 5-fold stratified cross-validation to ensure balanced outcome distribution across folds. Implementation was based on the tidymodels framework.

**
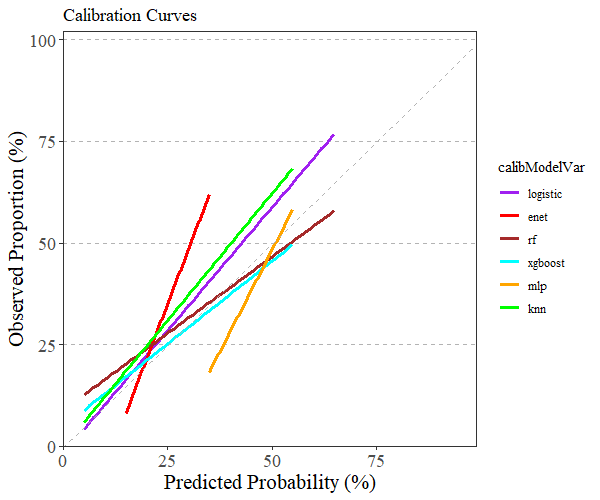
**

**Figure S1. Calibration plot.**
